# Supplementary material for: MiRNAs and mRNAs Analysis during Abdominal Preadipocyte Differentiation in Chickens
Source: Animals (Basel). 2020 Mar 11;10(3):468. doi: 10.3390/ani10030468 (PMC7143929; doi:10.3390/ani10030468)
Supplement: Supplementary file 1 [file animals-10-00468-s001.zip › Supplementary animals-731019/Supplementary Figures.docx]

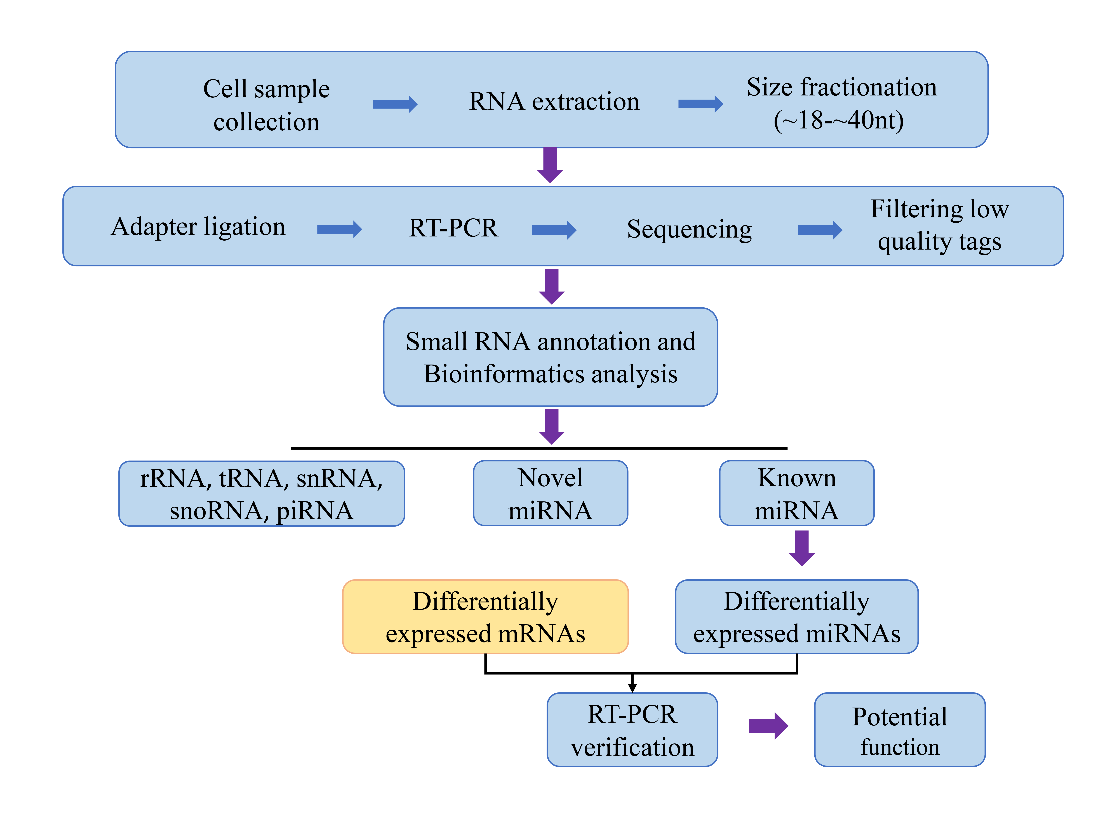


**Figure S1.** The Supplementary Figure S1 showed the overall idea of the experiment.


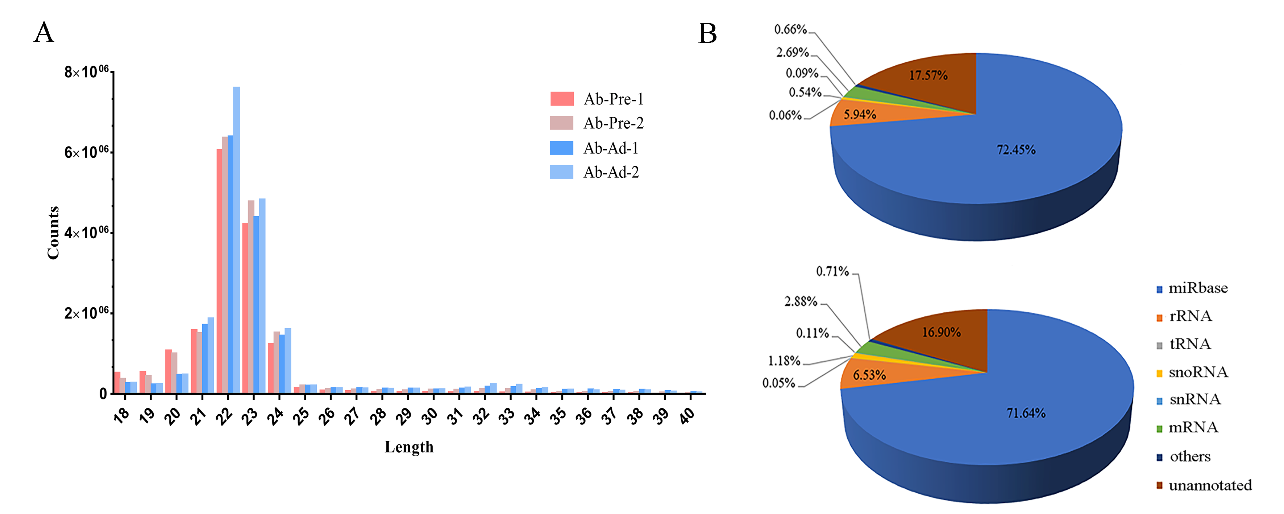


**Figure S2.** The Supplementary Figure S2 showed the length of small RNA sequence and the distribution of different sRNAs in adipocytes.


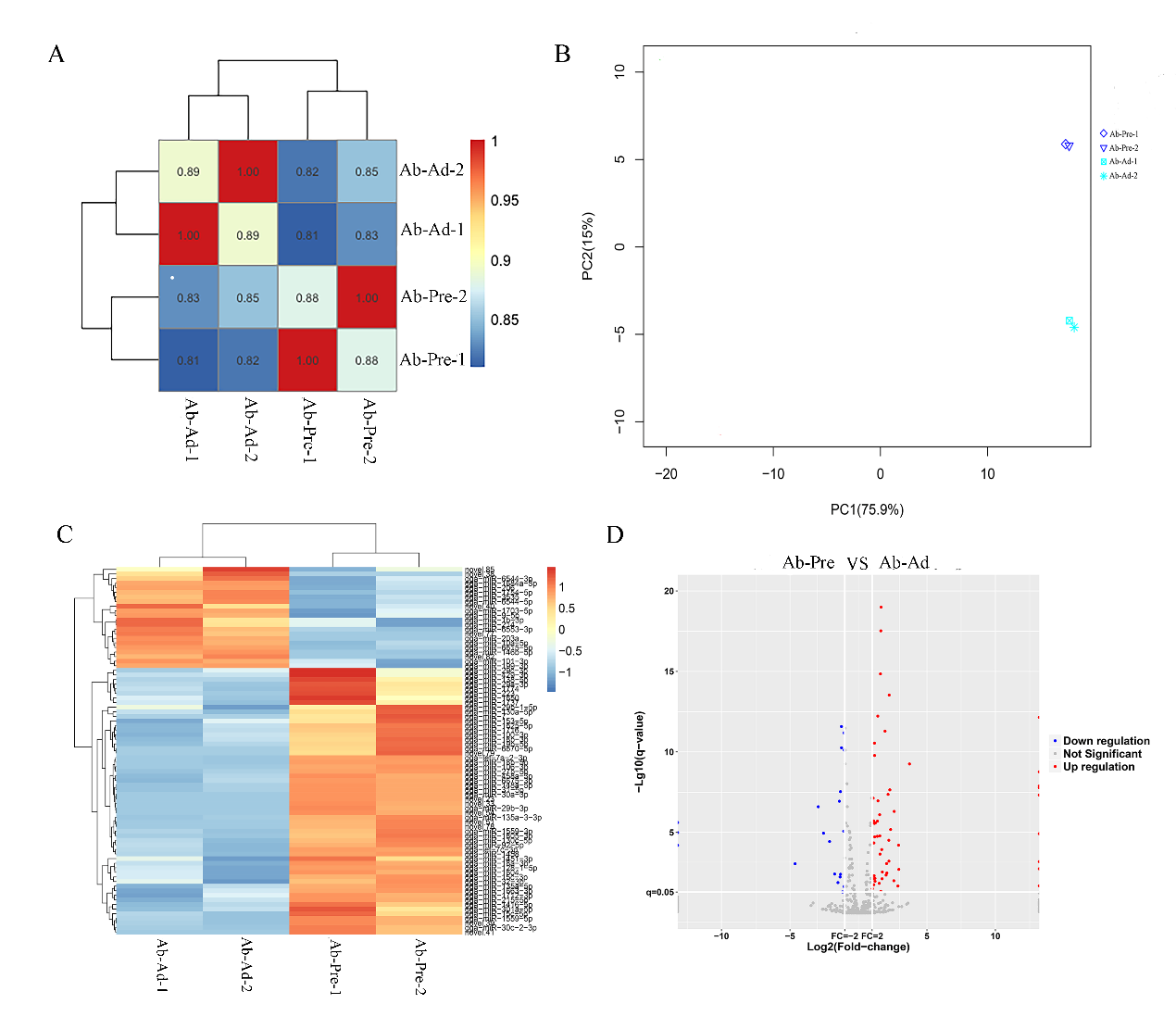


**Figure S3.** The Supplementary Figure S3 showed sample correlation, PCA, clustering and volcano map of diﬀerentiated miRNAs analysis between the preadipocytes (Ab-Pre) and differentiated adipocytes (Ab-Ad) groups.
