# Supplementary material for: MiRNAs and mRNAs Analysis during Abdominal Preadipocyte Differentiation in Chickens
Source: Animals (Basel). 2020 Mar 11;10(3):468. doi: 10.3390/ani10030468 (PMC7143929; doi:10.3390/ani10030468)
Supplement: Supplementary file 1 [file animals-10-00468-s001.zip › Supplementary animals-731019/Supplementary table S1.docx]

**Supplementary Table S1.** Specific qRT-PCR primers used in this study.

| **Gene name** | **Sequence** | **Product size (bp)** | **Accession NO.** |
| --- | --- | --- | --- |
| *ELOVL5* | F: 5’-CAGGAAGTCTCGAAACCGCT-3’  R: 5’-ACTCTGGGATCTCTGGGTCC-3’ | 193 | NM_001199197.1 |
| *COL6A1* | F: 5’-GAAAGGCACCCACACTGACT-3’  R: 5’-TGGCGACAGAGAACACTTTG-3’ | 200 | NM_205107.1 |
| *MAPK10* | F: 5’-TCTGCGCTTTGACGTCATGT-3’  R: 5’-TGGTTGCTGAATCACGCTCT-3’ | 154 | XM_420551.6 |
| *G6PC2* | F: 5’-GCTTTGGGCCTCAGTGGTAT-3’  R: 5’-TGACAAAAATCACAGTAATGCAACA-3’ | 299 | XM_025152633.1 |
| *ROCK2* | F: 5’-GGTACAGCCAAACCAGTCCA-3’  R: 5’-TCATGGAAAGCGGTTAGCCC-3’ | 193 | XM_015276085.2 |
| *β-actin* | F: 5’-CAGCCAGCCATGGATGATGA-3’  R: 5’-ACCAACCATCACACCCTGAT-3’ | 147 | NM_205518.1 |
